# Development and preliminary evaluation of the VPS ReplaySuite: a virtual double-headed microscope for pathology

**DOI:** 10.1186/1472-6947-5-10

**Published:** 2005-04-21

**Authors:** Dan J Johnston, Sean P Costello, Peter A Dervan, Daniel G O'Shea

**Affiliations:** 1 Medical Informatics Group, School of Biotechnology, Dublin City University, Dublin, Ireland; 2 The Conway Institute of Biomolecular and Biomedical Research, University College Dublin and The Pathology Department, Mater Misericordiae Hospital, Dublin, Ireland

**Keywords:** Telepathology, Virtual Slide, Pathology, Education, Quality Assurance, Double-Headed Microscope

## Abstract

**Background:**

Advances in computing and telecommunications have resulted in the availability of a range of online tools for use in pathology training and quality assurance. The majority focus on either enabling pathologists to examine and diagnose cases, or providing image archives that serve as reference material. Limited emphasis has been placed on analysing the diagnostic process used by pathologists to reach a diagnosis and using this as a resource for improving diagnostic performance.

**Methods:**

The ReplaySuite is an online pathology software tool that presents archived virtual slide examinations to pathologists in an accessible video-like format, similar to observing examinations with a double-headed microscope. Delivered through a customised web browser, it utilises PHP (Hypertext PreProcessor) to interact with a remote database and retrieve data describing virtual slide examinations, performed using the Virtual Pathology Slide (VPS).

To demonstrate the technology and conduct a preliminary evaluation of pathologists opinions on its potential application in pathology training and quality assurance, 70 pathologists were invited to use the application to review their own and other pathologists examinations of 10 needle-core breast biopsies and complete an electronic survey. 9 pathologists participated, and all subsequently completed an exit survey.

**Results:**

Of those who replayed an examination by another pathologist, 83.3% (5/6) agreed that replays provided an insight into the examining pathologists diagnosis and 33.3% (2/6) reconsidered their own diagnosis for at least one case. Of those who reconsidered their original diagnosis, all re-classified either concordant with group consensus or original glass slide diagnosis. 77.7% (7/9) of all participants, and all 3 participants who replayed more than 10 examinations stated the ReplaySuite to be of some or great benefit in pathology training and quality assurance.

**Conclusion:**

Participants conclude the ReplaySuite to be of some or of great potential benefit to pathology training and quality assurance and consider the ReplaySuite to be beneficial in evaluating the diagnostic trace of an examination. The ReplaySuite removes temporal and spatial issues that surround the use of double-headed microscopes by allowing examinations to be reviewed at different times and in different locations to the original examination. While the evaluation set was limited and potentially subject to bias, the response of participants was favourable. Further work is planned to determine whether use of the ReplaySuite can result in improved diagnostic ability.

## Background

Diagnostic pathology involves the classification of disease, using tissues obtained during biopsies, operations or autopsies. Classification is based on a complex set of visual features identified with the aid of a microscope. To reach a confident diagnosis, pathologists are required to possess well-developed searching, perception and classification skills, acquired with the assistance of intensive one-on-one tutoring sessions with expert pathologists. The workloads of expert pathologists, however, often restrict the frequency of these interactions.

While telepathology has yet to be incorporated into most pathology labs in a clinical role [1-4], advances in computing and telecommunications have resulted in the availability of a range of quality online training tools. Designed to supplement rather than replace human tutoring, such tools enable training pathologists to gain a wider range of educational experiences. Studies have shown the use of online training tools can improve diagnostic performance, beyond what is found with human tutoring alone [5,6].

The majority of training tools comprise interactive tutorials that display a limited number of pre-selected images per case, often with accompanying notation [7-15]. However, as has been observed through the use of static telepathology systems for remote diagnosis, diagnostic accuracy is often dependent on appropriate field selection [16-21]. Novices often make errors when searching the slide [22], so the development of the skills required to locate relevant visual features are as important as those required for identifying them. Neither the searching nor identification skills utilised to reach a diagnosis are assessed by conventional quality assurance studies; diagnostic accuracy is considered the principal indicator of competence.

In contrast to static systems, virtual slides (static-dynamic hybrids) enable unrestricted examination of entire digitised tissue sections [5,23-27]. This is a more accurate representation of the microscopic environment used by pathologists for diagnosis, and when incorporated into training tools, can provide unrestricted but supportive examining environments. With the cost and time required to digitise entire slides decreasing [28], virtual slides are finding greater application in pathology education [5,22,26].

In order to elucidate the diagnostic process of examining pathologists, the VPS (Virtual Pathology Slide), a virtual slide viewer that records diagnostic behaviour, has been developed and validated [23]. The VPS tracks each pathologist's slide examination, recording the location of, and time spent viewing each field, and storing the data on a remote, relational database. This data is available post-examination for interrogation, potentially enabling the diagnostic technique of different pathologists to be analysed and studied. While the VPS tracks user interactions with the software, it does not utilise this data constructively to provide an intrinsic benefit to the end user, the pathologist. For this purpose, the ReplaySuite was developed.

The ReplaySuite is a web-based, user-friendly software tool that enables pathologists to replay virtual slide examinations, performed using the Virtual Pathology Slide (VPS) [23,29,30]. Unlike interactive tutorials and annotated virtual slides, pathologists using the ReplaySuite are able to observe the diagnostic trace of examiners, in a manner similar to the use of a double-headed microscope. This possesses significant potential for use in both pathology training, where trainee pathologists may learn from the diagnostic techniques of experts, and quality assurance, for the detection and elucidation of sources of error in participants diagnostic technique. This paper describes the development and preliminary evaluation of the ReplaySuite technology and discusses its potential applications.

## Methods

### VPS

**Figure 1:**
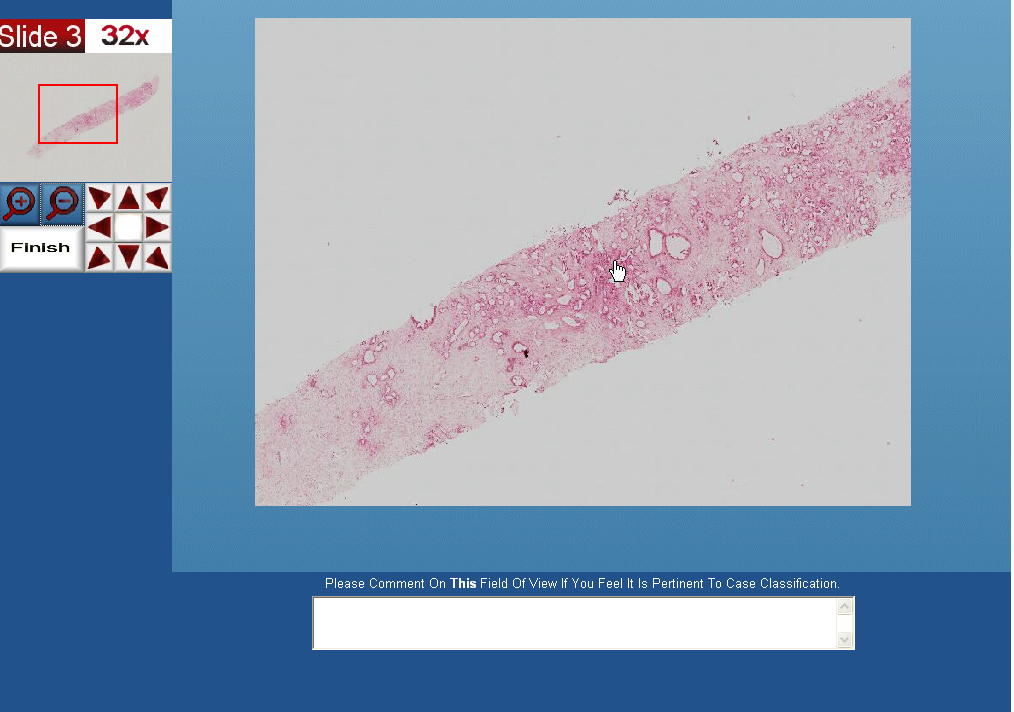
The VPS interface used by pathologist's when examining a slide.

The VPS is an interactive microscope emulator that presents a complete digitised tissue section via the Internet or CD ROM, allowing both stepwise increases of magnification (16× to 2000× on standard 17" monitor) and eight-way directional lateral-motion. During VPS examinations (Fig. 1), the locations of examined fields of view and the duration of time they were examined are recorded. While images are being retrieved from the remote server/CD ROM, examination data is transmitted back to the remote VPS server, where it is stored on a relational database management system (Oracle).

### Development of the ReplaySuite

The ReplaySuite is a web application that dynamically generates HTML (HyperText Markup Language) web pages to display images and text, using PHP, a server-side scripting language. It communicates with an Oracle database, on which all user tracking data is stored, via OCI (Oracle Call Interface) to retrieve and write data. JavaScript is used to deliver dynamic client side functionality within the graphical user interface (GUI). The ReplaySuite is deployed in a Microsoft Foundation Class (MFC) application written in Visual C++, which acts as a customised web browser. A complete description of the ReplaySuite specification is available in the ReplaySuite technical document [32].

Data describing an examination is retrieved from the remote server, via server side scripting, enabling communication between the application and the database. Structured Query Language (SQL) queries are embedded within PHP, interrogating the database and retrieving datasets. This data is used to determine which images to request from the server or CD-ROM, and how long to display these images. Server side scripting is also used to dynamically generate static HTML, which provides the GUI to display the images. During a replay, each image viewed during the examination is displayed in the order it was examined and for the duration it was examined. Diagnostic comments made during the examination are displayed adjacent to the appropriate field. Client side scripting (JavaScript) is used to dynamically alter the main field of view locator on the slide overview, indicate the number of seconds left until the next field of view is displayed and specify the upcoming action (increase/decrease magnification/lateral movement) performed to navigate to the next field.

### Using the ReplaySuite

**Figure 2:**
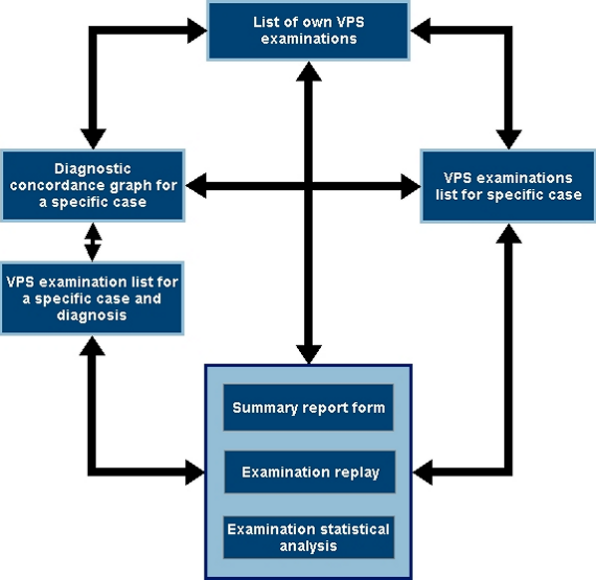
Flowchart of ReplaySuite operational functionality.

Users are required to login into the ReplaySuite, using a unique username and password. In order to maintain user anonymity, users are assigned unique VPS ID numbers. When examination or diagnostic data is displayed, these VPS ID numbers are used to identify the examining pathologist. Fig. 2 shows a flowchart of user navigation through available functionality when using the ReplaySuite. Users may navigate between lists of examinations, graphs of diagnostic concordance for examined cases and examination replays. During replays, users may return to the last viewed list of examinations, or their own examination list.

### Examination lists

**Figure 3:**
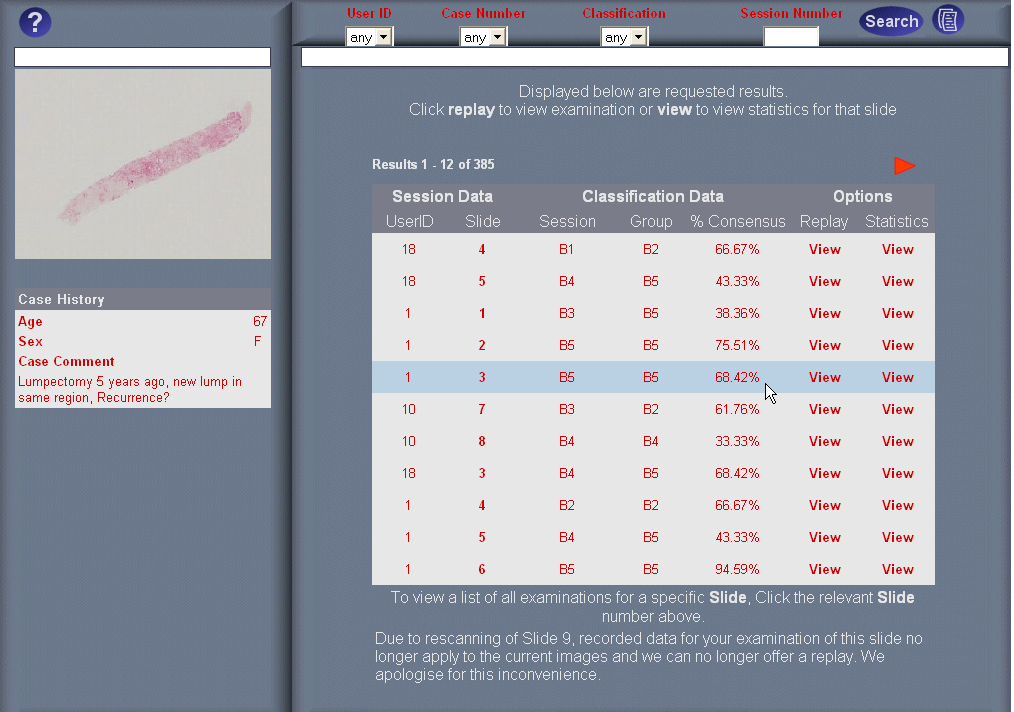
ReplaySuite list of examinations performed by User 6.

Upon logging in, the ReplaySuite displays an Examination list of all VPS examinations performed by that user. Users may also view, on demand, an Examination list of examinations for a specific case or sub-classify to view all examinations returning a specific diagnosis for a specific case. Examination lists display summarised information about each VPS examination, and for each examination displays, from left to right, the examining pathologists identification number (ID), the slide examined, the examiners diagnosis, group consensus diagnosis for the slide and the degree of group consensus (Fig 3). Highlighting an examination, within an Examination list, displays the slide overview and case history on the left of the interface. Examination lists are interactive and provide users with three options:

1. View an examination list of all examinations for a selected slide

2. View Diagnostic concordance graphs for a selected slide

3. Replay any users examination of a selected slide

### Diagnostic concordance graphs

**Figure 4:**
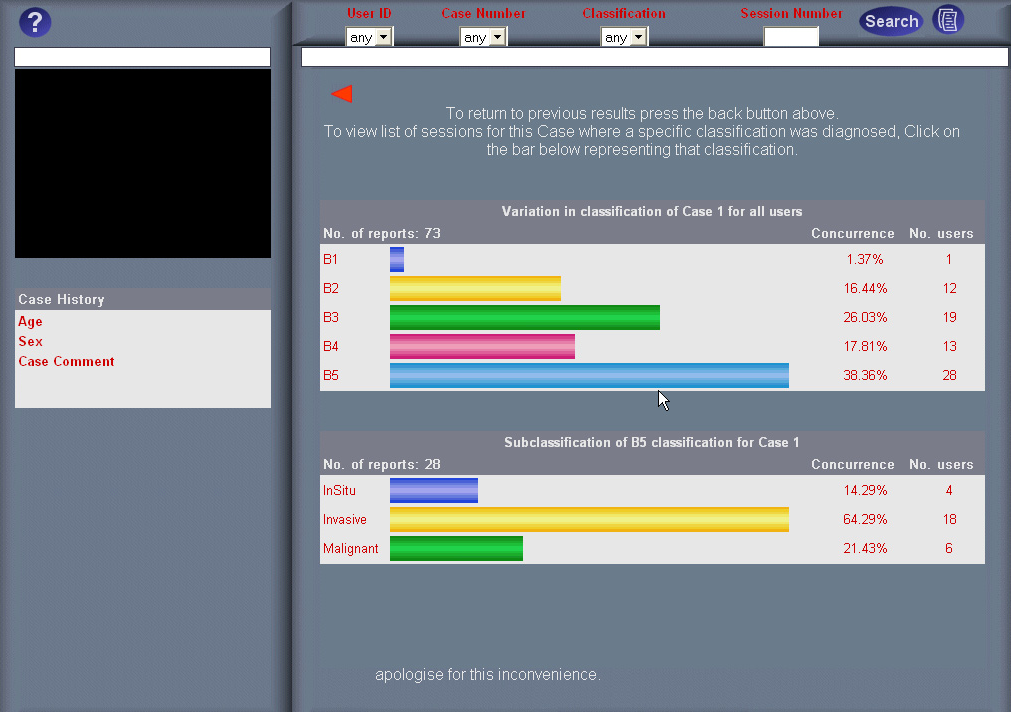
ReplaySuite variation in classification graph for slide 1.

Diagnostic concordance graphs are graphical representations of the variation in classification concluded by all examiners of a slide. Only data relating to slides previously examined by the user may be accessed. Two graphs are displayed; the top graph showing the variation in classification, the bottom graph the variation in sub-classification for B5 (malignant) diagnosis (Fig. 4). The number of examiners that concluded each diagnosis and the percentage concordance is also displayed in each graph. Clicking on any bar on either graph displays the list of all examinations of the selected slide that concluded with the selected classification/sub-classification. The ReplaySuite generates graphs by setting the dimensions of each bar according to the percentage of participants it represents, and can represent alternate scoring regimens by making simple changes to the system.

### Replaying an examination

**Figure 5:**
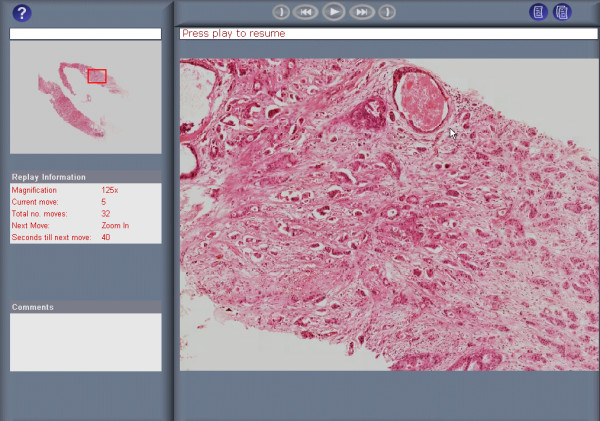
Replaying an examination of slide 2 using the ReplaySuite.

**Figure 6:**
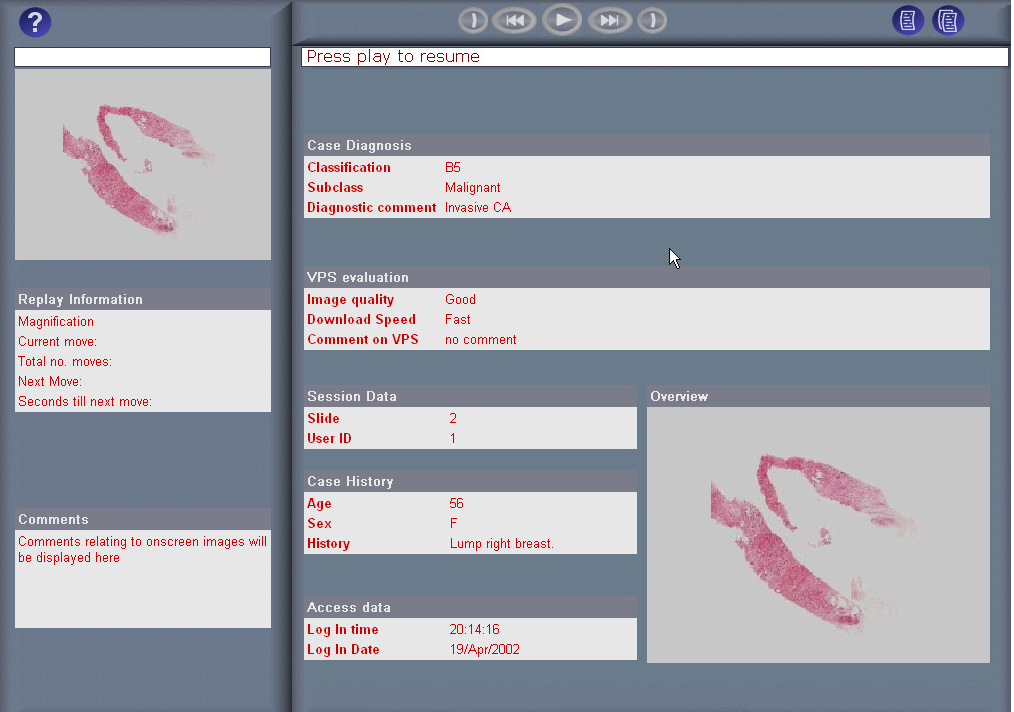
Summary report form from an examination replay using the ReplaySuite.

**Figure 7:**
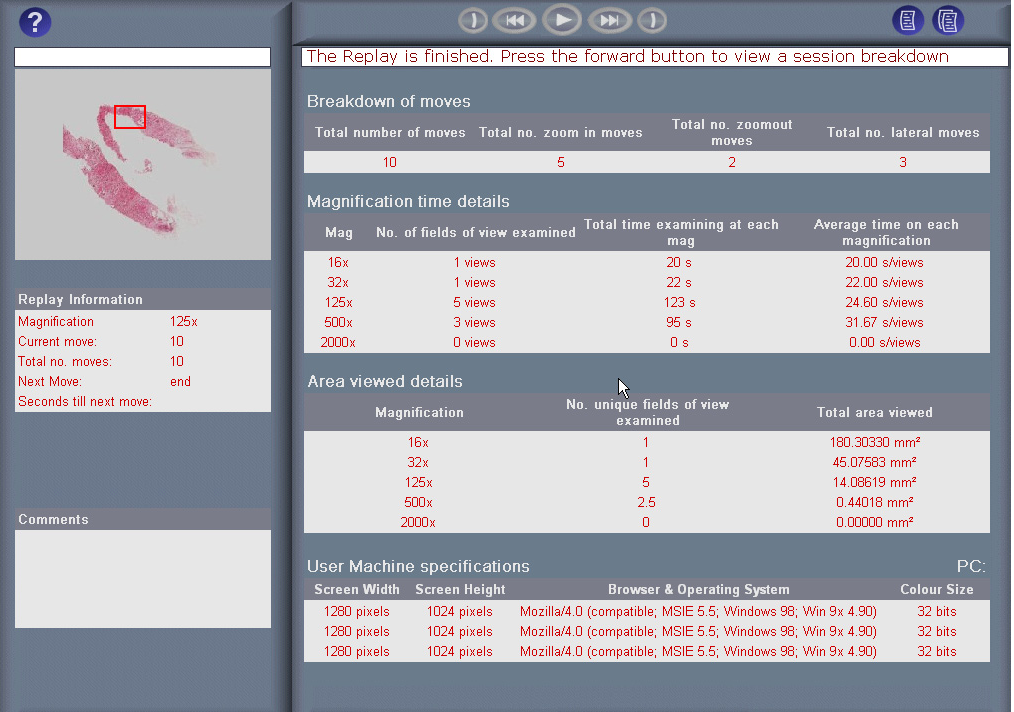
Statistical breakdown of a VPS examination using the ReplaySuite.

Clicking 'View' in the Replay column on the right of an Examination list replays an examination. During a replay, each field of view examined is displayed in chronological order, appearing on-screen for the duration it was originally examined. Unlike real-time observation of an examination, replays may be paused, fast-forwarded and rewound. A slide overview, located in the top left of the interface (Fig 5), relates the location of the magnified field of view to the tissue section, and notes made by the examining pathologist are displayed in the comment box. In addition, the summary report form submitted by the examining pathologist is displayed prior to a replay (Fig. 6), and a statistical analysis of the examination afterwards (Fig. 7). This statistical analysis displays the number and type of moves performed during the examination, the amount of time spent, number of fields viewed and area of tissue examined at each magnification and the specifications of the examining pathologists PC (screen resolution, colour depth and operating system).

### Preliminary evaluation of the ReplaySuite

#### Study procedure

A preliminary study was conducted to demonstrate the capabilities of the ReplaySuite to pathologists, and to evaluate their opinions on its use and potential application in training and quality assurance. Participants were provided with open access to the 10 needlecore biopsies examined during the VPS validation study [23], which they were required to examine using the VPS. Once a participant had submitted a diagnosis for a case, that participant was permitted to review their own examination data and that of other pathologists, for that slide, using the ReplaySuite. Participants who had previously participated in the VPS validation study (by examining cases) were not required to re-examine cases, and could review any examination.

Using the ReplaySuite, participants were permitted to view group concordance graphs, replay their own and others examinations, view summary report forms and view statistical breakdowns of individual examinations. Use of the ReplaySuite was monitored and recorded to the database, allowing the identification of light/heavy users and highlighting the most frequently used functions. Table 2 provides a summary of all participant use of the ReplaySuite. Cross-referencing VPS use (who examined which slides) with ReplaySuite use (who replayed which examinations) enabled the identification of which participants examinations were replayed and for which slides.

**Table 2:** Participant use of VPS and different functionality of ReplaySuite

	**VPS**	**ReplaySuite use**						
**User**	Examinations performed	Total examinations replayed	Own examinations replayed	Other participants examinations replayed	Diagnostic concordance graphs viewed	Examination lists viewed	**Total number of functions performed**	Survey Submitted

**10**	10	13	7	6	3	19	**35**	Y
**74**	10	12	4	8	11	35	**58**	Y
**55**	10	11	10	1	28	69	**108**	Y
**7**	10	3	2	1	4	12	**19**	Y
**36**	10	2	1	1	0	4	**6**	Y
**39**	10	2	2	0	13	9	**24**	Y
**102**	10	1	0	1	0	4	**5**	Y
**57**	10	0	0	0	9	4	**13**	Y
**60**	2	0	0	0	0	1	**1**	Y
**Total**	**92**	**44**	**26**	**18**	**68**	**157**	**269**	**9**

#### Slides

The ten needle core biopsies examined during the VPS validation study [23] were obtained by selecting the first breast biopsy generated each month for a ten month period from the Department of Pathology, Mater Misericordiae Hospital, Dublin, Ireland. Glass slide diagnosis (clinical diagnosis) was provided by a pathologist with a special interest in breast pathology using the Core Biopsy Reporting Guidelines for Non-operative Diagnostic Procedures and Reporting in Breast Cancer screening [31], as used by the British National Co-ordinating Committee for Breast Screening Pathology. Consultation was required to finalise glass slide diagnosis on some of the cases selected.

#### Participants

70 pathologists were invited to participate in the study. 38 Irish participants were invited with the assistance of Professor Peter Dervan, Mater Miscordiae Hospital, Dublin, and the remaining invitees comprising the 32 members of the European Working Group of Breast Screening Pathology (EWGBSP). EWGBSP members are recognised as expert breast pathologists in their native countries and all member states of the European Union (EU) are represented within the group. 17 of the 70 invited participants previously participated in the VPS validation study [23], 4 of which were members of the EWGBSP.

#### Exit survey

Subsequent to the study, participants completed an electronic questionnaire on their use and impressions of the software and its potential applications. Participants were asked to submit their level of agreement/disagreement with 19 statements using a Likert scale (5-point scale e.g. strongly disagree, disagree, neutral, agree, strongly agree), and answer Yes or No to a further 5 questions. A number of questions were inverted to mitigate against the well-known bias of positively phrased questions. The following are the pertinent questions asked:

### Where applicable answer yes or no to the following

• Did replaying an examination by another pathologist with a diagnosis different to your own provide you with an insight into why that diagnosis was concluded?

• Did replaying examinations performed by other pathologists make you reconsider your diagnosis for any slides?

### Please state how much you agree with the following statements

• The ReplaySuite is user-friendly

• The diagnostic pathway followed by the examining pathologist was apparent

• Being able to pause, fast-forward and rewind replays was useful

• The information panel was not useful

### Rate the benefit of using the ReplaySuite for the following applications

• Training

• Quality Assurance

Participants were also given the opportunity to provide open-ended feedback:

• Add any further comments, or have any additional features you would like to see incorporated into the ReplaySuite.

• If yes (to the question "Did you encounter technical difficulties while using the ReplaySuite?"), please state the difficulty that occurred

### Post study survey

Participants who used the ReplaySuite were resurveyed post-study in order to determine if they regularly participated in teaching. 3 questions were asked:

• Are you, or have you been involved in providing undergraduate medical training on a regular basis?

• Are you, or have you been involved in providing postgraduate pathology training on a regular basis?

• If you are not currently involved but have previously been in either activity, please state how long ago you were involved.

## Results

### Study participation

9 participants used the ReplaySuite to review examination data. All 9 completed the electronic questionnaire, and all but one had more than five years experience in pathology practice, the exception possessing three years experience. 4 of the 9 participants were members of the European Working Group of Breast Screening Pathology. Table 1 summarises study participation. All 9 participants completed the post-study survey.

**Table 1:** User participation in evaluation of ReplaySuite technology

	**Irish**	**EWGBSP**	**Total**
**Invited to participate**	38	32	70
**Used ReplaySuite **	5	4	9
**Completed electronic survey**	5	4	9

### Using the ReplaySuite

**Figure 8:**
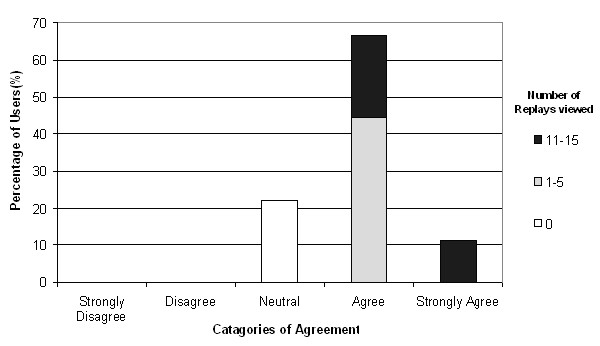
Levels of agreement with the statement 'The ReplaySuite is User-Friendly'.

7 participants (77.7%) replayed at least one examination and of these, 3 participants replayed more than 10. All participants who replayed at least one examination agreed or strongly agreed that the ReplaySuite was user-friendly (Fig. 8) and 85.71% (6/7) of these agreed or strongly agreed that being able to pause, fast-forward and rewind an examination replay was useful. 6 of the 7 of participants (85.71%) who replayed an examination considered replays to be of some or of great importance as a ReplaySuite feature. 66.6% (6/9) of all participants viewed diagnostic concordance graphs for at least 2 slides and 2 participants (44.4%) viewed graphs for at least 7 slides.

### Diagnostic re-evaluation

All participants who replayed at least one examination agreed or strongly agreed that the diagnostic pathway of the examining pathologist was apparent during a replay. Of the 7 participants who replayed their own or others examinations, 6 (85.71%) replayed another pathologists examination. Of these 6 participants, 5 (83.3%) agreed that it provided an insight into the examining pathologists diagnosis. Of those who replayed another's examination, 2 (33.3%) reconsidered their original diagnosis, 1 re-diagnosing concordant with group consensus and 1 re-diagnosing concordant with original glass slide diagnosis.

After originally concluding an original diagnosis of B2 (Benign) for Slide 5, User 7 replayed his/her own examination, and then replayed an examination of Slide 5 by User 6. This examination (User 6) concluded a diagnosis of B5 (Malignant), concordant with group consensus.

User 10 concluded an original diagnosis for Slide 8 of B4 (Suspicion of malignancy), which concurred with group consensus but not glass slide diagnosis, B3 (Benign but of uncertain malignant potential). User 10 replayed their own examination, then an examination of the same slide that concluded the same diagnosis by User 5. Two examinations concluding a B3 diagnosis were then replayed (Users 87 and 55), concordant with glass slide diagnosis.

### ReplaySuite applications

**Figure 9:**
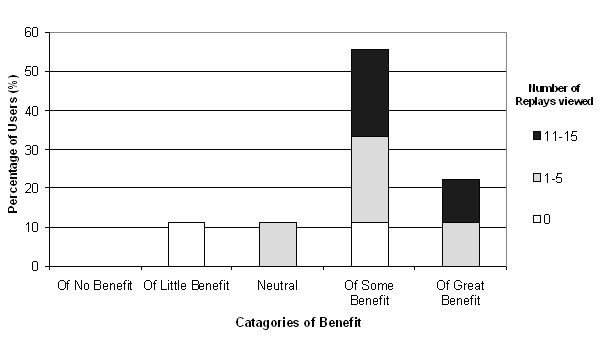
Levels of perceived potential benefit of the ReplaySuite in Pathology Training.

When asked to rate the potential benefit of the ReplaySuite in pathology training, 6 out of 7 of participants who had replayed at least one examination (85.71%) and all of those who had replayed more than 10 examinations rated it as of some or of great benefit (Fig. 9). Of the 9 that completed the post-study survey, 7 and 8 stated they were currently, or had been involved in providing undergraduate medical training and postgraduate pathology training on a regular basis respectively.

**Figure 10:**
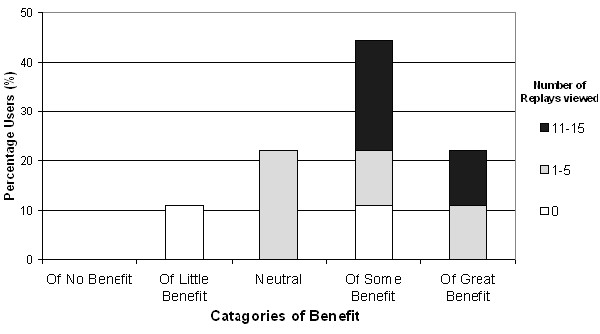
Levels of perceived potential benefit of the ReplaySuite in Pathology Quality Assurance.

When asked to rate the potential benefit of the ReplaySuite in quality assurance, 5 out of 7 of participants who had replayed at least one examination (71.4%) and all participants who had replayed more than 10 examinations considered it of some or of great benefit (Fig. 10).

## Discussion

The VPS tracks users examinations of VPS virtual slides, however, in isolation this data does not directly benefit the end user. The ReplaySuite was developed to exploit the VPS's examination tracking capabilities and utilise the data in a manner that would have tangible benefits for pathologists. Using a combination of server-side (PHP) and client-side (JavaScript) open source scripting languages, it presents this data to users via an intuitive Graphical User Interface (GUI), which was considered easy to use by participants who replayed at least 1 examination (Fig. 8). Utilising the same technology as the VPS, the ReplaySuite does not require the installation of any additional software other than the customised browser, and Internet Explorer, which is the default web browser used by 95% of the web browser market [33].

Use of the VPS for diagnosis has already demonstrated "substantial" diagnostic agreement between users, 88.23% of examining pathologists obtaining a Kappa of between 0.97 and 0.65 [23]. In determining whether pathologists could benefit from using the ReplaySuite, the primary consideration was whether participants might reassess their diagnosis as a result of observing VPS examinations of the same slides by others. In the case of 2 users, observing another's examination caused them to reconsider their original diagnosis. In contrast to the re-diagnosis of Slide 8 by User 10: B4 (suspicion of malignancy) to B3 (Benign but of uncertain malignant potential), the difference between original and secondary diagnosis of Slide 5 by User 7: B2 (benign) to B5 (malignant) is a significant re-evaluation, one which would result in significantly different courses of treatment in a clinical environment. While the degree of discordance between original diagnosis and group consensus may, in part be attributable to poor screen resolution used during examination (640 × 480 pixels), it may also be related to the relative difficulty of the case; Slide 5 achieved the second lowest group consensus (47.1%). It cannot be suggested, based on these individual examples, that group concordant re-diagnosis subsequent to ReplaySuite use will be the rule rather than the exception. However, these examples are worth noting, as it highlights the fact that using the system can result in diagnostic re-evaluation.

A number of caveats should be considered when reviewing study data, the first being small sample size. There is a significant difference between the number of pathologists invited to participate and those who completed the study (9/70). While not unique in studies involving pathologists use of telepathology systems, the small sample size may be considered a potential source of error. Bamford *et al *(2003) have reported similar difficulties with low participation rates [34], however, other studies have also attempted to evaluate telepathology tools using small sample sizes [35]. Low participation rates may be due to a number of factors. Bamford *et al *(2003) cited technical difficulties and pathologists workloads as principal factors for low participation [34]. System speed was highlighted as an issue by a number of participants during the initial VPS evaluation study [23]. As both VPS and ReplaySuite systems utilise similar technology, speed may be a potential contributing factor to low participation rates for this follow-on study. User 36 illustrated this when asked to comment on any technical difficulties encountered during the ReplaySuite evaluation study, stating, "It took a great deal of time downloading image via dial up network connection". User 60 also commented to this effect regarding technical difficulties, commenting "very slow".

Secondly, it is not unreasonable to suggest that the positive evaluation of the ReplaySuite may in part be attributable to bias from participants (4/9) who also previously participated in the VPS validation study. Pathologists who participated in both VPS and ReplaySuite studies expressed greater satisfaction and confidence in the VPS during the VPS validation study than pathologists who participated only in the VPS study [23]. However during the ReplaySuite study, these participants who participated in both did not provide more favourable evaluation of the ReplaySuite than participants who only participated in the ReplaySuite study. In Figure 8, the participant who considered the ReplaySuite the most user-friendly did not participate in the VPS evaluation study. In Figure 9, all participants who considered the ReplaySuite of greatest benefit in training were participants who did not participate in the VPS evaluation study, and both participants and non-participants in the VPS evaluation study considered the ReplaySuite of great benefit in quality assurance studies (Figure 10).

It also goes without question that those who participate in studies of this nature are often early adapters and often possess a positive bias towards new technology. This is an issue when evaluating any new software, and in that context, bias is unavoidable. 20 of the 70 participants invited to participate had foreknowledge of the VPS, however none, had previously seen the ReplaySuite and, therefore, had no preconceived notions about the software.

The concept of a virtual double-headed microscope is not new [36], however previous references to such concepts utilised video-conferencing that was dependent on the presence of an expert. While both pathologists were not required to be present at the same location, they were required to be available at the same time. In contrast, archived VPS examinations are available at any time at any location, irrespective of expert availability. Unlike real-time observation of examinations, the ReplaySuite permits examinations to be paused, fast-forwarded and rewound, and images may be annotated, providing a description of visual features within the field of view.

The benefits of various online tools and their impact on diagnostic performance have already been highlighted, however the ReplaySuite possesses a number of practical advantages. Many online tools present the diagnostic opinion of one pathologist. In contrast, the ReplaySuite can replay examinations of the same slide by multiple experts, illustrating a number of different diagnostic pathways that corroborate the same diagnostic hypothesis. Alternatively, reviewing pathologists may observe examinations that disagreed with group consensus, in order to identify the possible sources of disagreement. This is of particular interest for disorders that suffer from a high degree of inter-observer variability. Additionally, interactive tutorials and annotated virtual slides require considerable time to create, however, individual authoring time with the VPS/ReplaySuite is around 6 minutes, per user, per slide.

Figures 9 &10 illustrate that the more participants used the system, the greater potential benefit they perceived it having in pathology training and quality assurance. All 3 participants (Users 10,55,74) who replayed more than ten examinations considered the ReplaySuite of some or of great potential benefit in pathology training and quality assurance. While small sample size precludes the significance of a relationship between heavy use and favourable perception, it is not unreasonable to suggest that participants who fully appreciate the capabilities of the ReplaySuite will possess a more considered opinion.

## Conclusion

The objective of the study was to develop the ReplaySuite technology, and to assess the opinions of pathologists on its use and potential application. Participants concluded the ReplaySuite to be of some or of great potential benefit to pathology training and quality assurance and considered the ReplaySuite to be beneficial in evaluating the diagnostic trace of an examination. Future evaluation of the ReplaySuite in a larger quality assurance study and training environment is anticipated.

One-to-one human tutoring remains, and will remain for the foreseeable future, the pre-eminent means of nurturing pathologist's diagnostic skills. However, as factors such as expert availability and case diversity vary, from institution to institution, it is impossible to replicate the same training environment universally. Supplementary online training tools may well help to redress the balance, providing wide access to expert pathologists and a diverse range of cases from multiple institutions, enabling an educational diversity often unachievable in a single institution. The ReplaySuite is a unique tool that permits pathologists to learn from multiple experts in a manner not possible with current tools.

## Competing interests

DS and SC possess share holdings in Slidepath Ltd, a company delivering informatics solutions for use in pathology quality assurance. DJ and PD have share options in SlidePath Ltd.

## Authors' contributions

DS conceived the ReplaySuite and participated in the design of the study. DJ created the ReplaySuite, carried out the study and drafted the manuscript. SC created the VPS and participated in the design of the database. PD participated in the design of the ReplaySuite and provided a pathologist's insight.

## Pre-publication history

The pre-publication history for this paper can be accessed here:


